# Transfer of the Resistance to Multiple Diseases from a *Triticum*-*Secale*-*Thinopyrum* Trigeneric Hybrid to Ningmai 13 and Yangmai 23 Wheat Using Specific Molecular Markers and GISH

**DOI:** 10.3390/genes13122345

**Published:** 2022-12-12

**Authors:** Yi Dai, Juntao Shi, Jinfeng Li, Yujiao Gao, Haigang Ma, Yonggang Wang, Baotong Wang, Jianmin Chen, Peng Cheng, Hongxiang Ma

**Affiliations:** 1 Joint International Research Laboratory of Agriculture and Agri-Product Safety, The Ministry of Education of China/Jiangsu Key Laboratory of Crop Genomics and Molecular Breeding/Jiangsu Co-Innovation Center of Modern Production Technology of Grain Crops, Yangzhou University, Yangzhou 225009, China; 2 State Key Laboratory of Crop Stress Biology for Arid Areas, College of Plant Protection, Northwest A&F University, Yangling 712100, China

**Keywords:** rye, *Thinopyrum elongatum*, multiple disease resistance, powdery mildew, stripe rust, FHB

## Abstract

The middle to lower reaches of the Yangtze River are China’s second largest area for wheat production; wheat disease is more serious there than in other areas because of the high humidity and warm weather. However, most cultivated varieties are susceptible to Fusarium head blight (FHB), powdery mildew, and stripe rust, and the lack of disease-resistant germplasm is an obstacle in wheat breeding. Rye and *Thinopyrum elongatum*, related species of wheat, carry many genes involved in disease resistance. In this study, a trigeneric hybrid, YZU21, with resistance to FHB, powdery mildew, and stripe rust was used to improve two major wheat cultivars, Ningmai 13 (NM13) and Yangmai 23 (YM23). Specific molecular markers and GISH were used to identify hybrid progenies. Five addition or substitution lines and one translocation line of the *Triticum-Secale-Thinopyrum* trigeneric hybrid were obtained and evaluated for agronomic traits and the resistance to multiple diseases. The results showed that the six trigeneric hybrid lines had desirable agronomic traits and improved resistance to FHB, powdery mildew, and stripe rust; they might be used as parents in wheat breeding for the resistance to multiple disease.

## 1. Introduction

Wheat is one of the earliest domesticated crops and has been a staple food for thousands of years. Common wheat is an allohexaploid species composed of genomes A, B, and D. It originated from two successive rounds of polyploidization within the genera *Triticum* and *Aegilops*, forming tetraploid wheat (AABB) and hexaploid wheat (AABBDD) [1]. Although polyploidy through the fusion of genomes from different environments broadens the adaptability of wheat, this process severely reduces genetic diversity because of domestication bottlenecks. In addition, the selection of agronomic traits has led to a universal convergence of wheat adaptations worldwide. Most cultivated varieties can hardly cope with biotic and abiotic stress, which seriously affects wheat development. Therefore, the creation and enrichment of wheat germplasm resources are important for broadening the genetic diversity of wheat. The gene pool of wild related species in wheat retains many excellent characteristics that common wheat lacks in natural evolution, such as disease resistance, insect resistance, drought tolerance, and saline-alkali tolerance. Introducing these genes into common wheat can enrich the genetic diversity of wheat and is of great significance for wheat genetic improvement.

Rye (*Secale graal* L., 2n = 2x = 14, RR) is an important food crop in the middle-, eastern- and northern-latitude countries. Compared with wheat and other small grain crops, rye is the cereal with the highest tolerance to drought, salt, and aluminum stress; it also carries many disease resistance genes [2], such as the powdery mildew resistance genes *Pm7* [3], *Pm8* [4], *Pm17* [4], *Pm20* [5], and *Pm56* [6]; the stem rust resistance genes *Sr27* [7], *Sr31* [8], *Sr50* [9], and *Sr59* [10]; the leaf rust resistance genes *Lr25* [3], *Lr26* [8], and *Lr45* [11]; and the stripe rust resistance gene *Yr9* [8]. In the past 10 years, some new wheat germplasms carrying 1RS chromosomes have shown resistance to powdery mildew (*Blumeria graminis* f. sp. *tritici*, *Bgt*) and stripe rust (*Puccinia striiformis* f. sp. *tritici*, *Pst*) pathogens and have been widely used in wheat breeding [12]. In addition, the breeders also successfully transferred rye chromosomes to wheat through artificial synthesis, creating an artificial synthetic crop, triticale, which can be divided into tetraploid (AARR, 2n = 4x = 28), hexaploid (AABBRR, 2n = 6x = 42), octaploid (AABBDDRR, 2n = 8x = 56), and decaploid (AABBDDRRR, 2n = 10x = 70) triticale, based on different chromosome compositions and ploidy. As a crop, triticale has many excellent characteristics, such as high resistance to powdery mildew, yellow dwarf, leaf rust, and other diseases. As a food, it can also regulate the absorption of nutrients, improve immunity, and control blood sugar levels, thus reducing the risk of some common diseases such as cardiovascular disease and obesity, which is greatly beneficial to human health [13]. Unfortunately, triticale is not as resistant to Fusarium head blight (FHB) as wheat [14]. Therefore, improving the resistance to FHB has become the main direction for triticale variety improvement.

*Th. elongatum* is a closely related wild species of wheat, and the E genome has relatively little genetic differentiation from the A, B, and D genomes of wheat; thus, *Th. elongatum* is a valuable exogenous genetic resource for wheat. It was found that *Th. elongatum* has excellent resistance to FHB. For example, Jauhar et al. [15] found that durum wheat-*Th. elongatum* 1E additional line, 1E (1A), and 1E (1B) diploid substitution lines were highly resistant to FHB, suggesting that the 1E chromosome of diploid *Th. elongatum* may harbor FHB resistance genes. Shen and Ohm [16] found that the 7E chromosome of the diploid *Th. elongatum* may harbor FHB resistance genes. In addition, Liu et al. [17] found that the FHB resistance gene was present on the 7E chromosome of tetraploid *Th. elongatum* by constructing a durum wheat–*Th. elongatum* 7E additional line. Guo et al. [18] localized an FHB resistance QTL on chromosome 7el_2_ of *Th. ponticum* and was named *Fhb7*. Later, Wang et al. [19] successfully cloned the *Fhb7* gene from *Th. ponticum* and demonstrated that this gene encodes glutathione S-transferase (GST), which catalyzes the formation of DON-GSH from DON toxin, thus acting as a detoxifier and enhancing FHB resistance in wheat. Therefore, the transfer of *Th. elongatum* chromosomes or chromosome fragments carrying the FHB resistance gene into triticale can improve the FHB resistance of triticale. For example, Dai et al. [20] crossed hexaploid triticale T182 (AABBRR) with hexaploid *Triticum. trititrigia* 8801 (AABBEE) to create a *Triticum-Secale-Thinopyrum* trigeneric hybrid RE21, which was highly resistant to leaf rust, stem rust race Ug99, and FHB.

China is the largest producer and consumer of wheat in the world. The region of the middle and lower reaches of the Yangtze River is China’s second largest wheat belt. It has great potential for wheat production because it is a rainfed agricultural region, and the production cost is relatively lower than that in the northern winter wheat area. However, owing to the high humidity and warm weather, wheat diseases, such as FHB and powdery mildew, are more serious than in the northern winter wheat region. In recent years, wheat stripe rust has also frequently occurred because of climate change. Ningmai 13 (NM13) and Yangmai 23 (YM23) are major cultivars grown in the middle and lower reaches of the Yangtze River, which have high yield potential and good quality; however, their resistance to FHB, powdery mildew, and stripe rust needs to be further improved.

In this study, wheat cultivars NM13 and YM23 were crossed with YZU21, a derivative of *Triticum-Secale-Thinopyrum* trigeneric hybrid RE21 [20], to improve resistance to FHB, powdery mildew, and stripe rust. Specific molecular markers, genome in situ hybridization (GISH), were used to identify hybrid progeny. Agronomic traits and resistance to FHB, powdery mildew, and stripe rust were also evaluated. Six lines of trigeneric hybrids with desirable agronomic traits and improved resistance to FHB, powdery mildew, and stripe rust were obtained, which could be used as parents in wheat breeding for improving resistance against multiple diseases.

## 2. Materials and Methods

### 2.1. Plant Materials

The *Triticum-Secale-Thinopyrum* trigeneric hybrid RE21 was developed by Dai et al. [20], it carried alien chromosomes 1E, 2E, 3E, and 5E from tetraploid *Th. Elongatum* and chromosomes 4R, 6R, and 7R from rye, and has excellent resistance to leaf rust, stem rust race Ug99, and FHB. Since 2016, RE21 has been planted at Yangzhou University. Individual plants with excellent agronomic traits that adapted to the climate conditions in the middle and lower reaches of the Yangtze River were selected and planted into a line named YZU21. The tetraploid *Th. elongatum* (2n = 4x = 14, E^e^E^e^E^b^E^b^), hexaploid triticale T182 (2n = 6x = 42, AABBRR), and hexaploid *T. trititrigia* 8801 (2n = 6x = 42, AABBEE) were provided by Professor George Fedak, retired from the Ottawa Research and Development Center, Agriculture and Agri-Food Canada. Ningmai 23 (NM13) and Yangmai 23 (YM23) are the main cultivated wheat varieties in the middle and lower reaches of the Yangtze River, China. YZU21 was crossed directly with NM13 and YM23 to produce hybrid F_1_ seeds. Some F_1_ plants were backcrossed with wheat parent NM13 and YM23 once to produce BC_1_F_1_ plants, and the remaining plants were selfed to produce hybrid F_2_ seeds. To date, only plants with excellent agronomic traits and carrying the E and R chromosomes have been harvested. In generations F_5_ to F_6_ and BC_1_F_3_ to BC_1_F_4_, the chromosome composition of the plants was examined by GISH and disease resistance identification. Finally, new wheat lines with stable agronomic traits and disease resistance were identified.

### 2.2. Molecular Marker Analysis

In generations F_1_ to F_6_ and BC_1_F_1_ to BC_1_F_4_, E and R genomic markers were used to identify alien chromosomes. From generations F_5_ and BC_1_F_3_, the chromosome-specific markers were used to identify chromosome composition. PCR amplification was carried out in a 25 μL reaction containing 1 μL genomic DNA (100 ng/μL), 12.5 μL 2× Taq master mix (Vazyme, P112-AA), 1 μL each of primers, and 9.5 μL double-distilled water. The primer sequences are listed in Table S1. The PCR procedure was as follows: 95 °C for 5 min, followed by 36 cycles of 95 °C for 30 s, an appropriate annealing temperature (50–65 °C) for 45 s, 72 °C for 40 s, and a final extension for 10 min at 72 °C. Amplified products were electrophoresed on a 1% agarose gel in 1× TAE buffer.

### 2.3. Cytogenetic Analyses

Chromosomes were prepared from mitotic metaphase cells following the protocol described by Lei et al. [21], and GISH analysis was followed by the methods described by Zhao et al. [22]. Total genomic DNA of Imperial rye and tetraploid *Th. elongatum* were labeled using DIG-Nick Translation Mix (Roche, Mannheim, Germany) and Biotin-Nick Translation Mix (Roche), respectively, for use as probes for multicolor GISH. The genomic DNA of Chinese Spring was used as blocking DNA. Hybridization signals were observed using a fluorescent microscope, and images were obtained using a CCD camera (Color Cooled Digital DS-Fi1c, Nikon 80i; Nikon, Tokyo, Japan). Images were processed using Photoshop CC (Adobe Systems, Inc., San Jose, CA, USA).

### 2.4. Evaluation of Disease Resistance

Four *Bgt* races, E09, E15, A13, and A44, were used in the powdery mildew resistance tests at the seedling stage. Two *Pst* races, CYR32 and CYR34, were used in this study. *Bgt* and *Pst* were inoculated on wheat seedlings according to the methods described by Yang et al. [23] and Ren et al. [24]. At the seedling stage, powdery mildew reactions were scored as ITs based on a 0–4 scale, as described by Xie et al. [25]. Stripe rust reactions were scored as infection types (ITs) based on a 0–9 scale, as described by Ren et al. [12].

## 3. Results

### 3.1. Creating New Breeding Lines Carrying Alien Chromosomes from Trigeneric Hybrid Line YZU21

**Figure 1:**
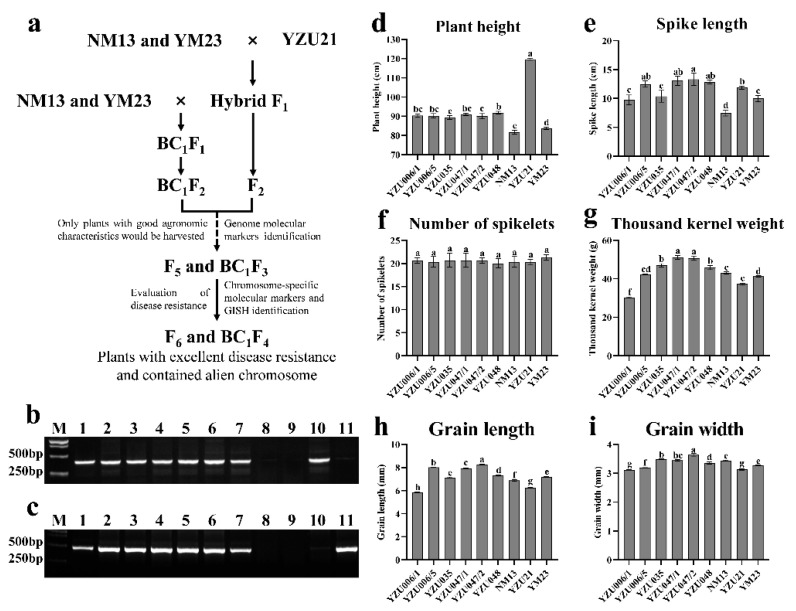
The PCR identification and investigation of field agronomic characters of new breeding lines. (**a**) The pedigree of the new breeding lines; (**b**,**c**) PCR results of R and E genomic molecular markers, respectively. M: marker, lane 1: YZU006/1, lane 2: YZU006/5, lane 3: YZU035, lane 4: YZU047/1, lane 5: YZU047/2, lane 6: YZU048, lane 7: YZU21, lane 8: NM13, lane 9: YM23, lane 10: T182, lane 11: 8801. Statistical analysis of (**d**) plant height, (**e**) spike length, (**f**) number of spikelets, (**g**) thousand kernel weight, (**h**) grain length, and (**i**) grain width. The data were statistically analyzed via Kruskal–Wallis one-way ANOVA. Pairwise comparisons were completed using LSD. Different letters show significance at *p* < 0.05.

To transfer diseases resistance to common wheat, YZU21 was crossed as male parent with the common wheat varieties NM13 and YM23. Two or three spikes from each F_1_ plant were selected and backcrossed with NM13 and YM23, whereas the rest of the spikes were allowed to self-pollinate to obtain offspring seeds. Based on the results of whole genome molecular marker analysis of *Th. elongatum* and rye, and the field evaluation of agronomic characters, six lines with stable agronomic characters were obtained (Figure 1a and Table 1). The six lines contained the chromosomes of *Th. elongatum* and rye (Figure 1b,c), and spike length, grain weight, grain length, and grain width were better than those of NM13 and YM23. Among them, the spike lengths of YZU006/5, YZU047/1, YZU047/2, and YZU048 were significantly longer than those NM13 and YM23 (Figure 1e); and the grain weights of YZU035, YZU047/1, YZU047/2, and YZU048 was higher than those of NM13 and YM23 (Figure 1g). The grain length and width of YZU047/1 and YZU047/2 were better than those of NM13 and YM23 (Figure 1h,i). Therefore, these new breeding lines can be used as intermediate for improving wheat yield.

### 3.2. Individual Alien Chromosome Identification of New Breeding Lines Using Chromosome-Specific Markers

**Figure 2:**
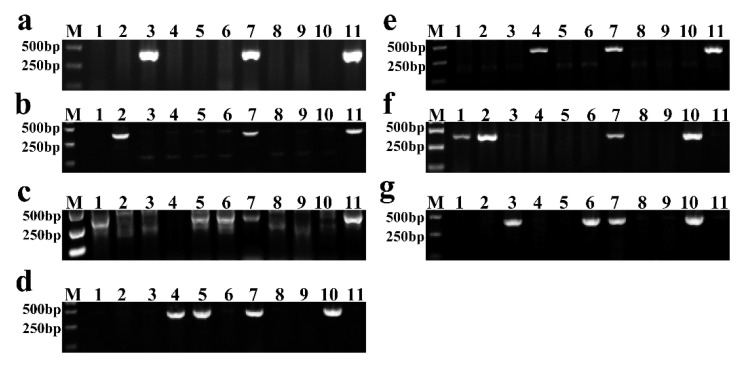
The PCR identification of new breeding lines by using chromosome-specific markers. (**a**–**g**) The PCR results of 1E, 2E, 3E, 4R, 5E, 6R and 7R chromosome-specific markers, respectively. M: marker, lane 1: YZU006/1, lane 2: YZU006/5, lane 3: YZU035, lane 4: YZU047/1, lane 5: YZU047/2, lane 6: YZU048, lane 7: YZU21, lane 8: NM13, lane 9: YM23, lane 10: T182, lane 11: 8801.

This study used 1E, 2E, 3E, 4R, 5E, 6R, and 7R chromosome-specific molecular markers to identify individual alien chromosomes in new lines. The PCR results showed that YZU035 carried 1E chromosome (Figure 2a); YZU006/5 carried the 2E chromosome (Figure 2b); three lines, YZU006/1, YZU047/2 and YZU048 contained the 3E chromosome (Figure 2c); YZU047/1 and YZU047/2 all carried the 4R chromosome (Figure 2d); YZU047/1 carried the 5E chromosome (Figure 2e); YZU006/1 and YZU006/5 contained 6R chromosome (Figure 2f); and YZU035 and YZU048 carried 7R chromosomes (Figure 2g).

### 3.3. Chromosome Constitution Analysis of New Breeding Lines by Multicolor GISH

**Figure 3:**
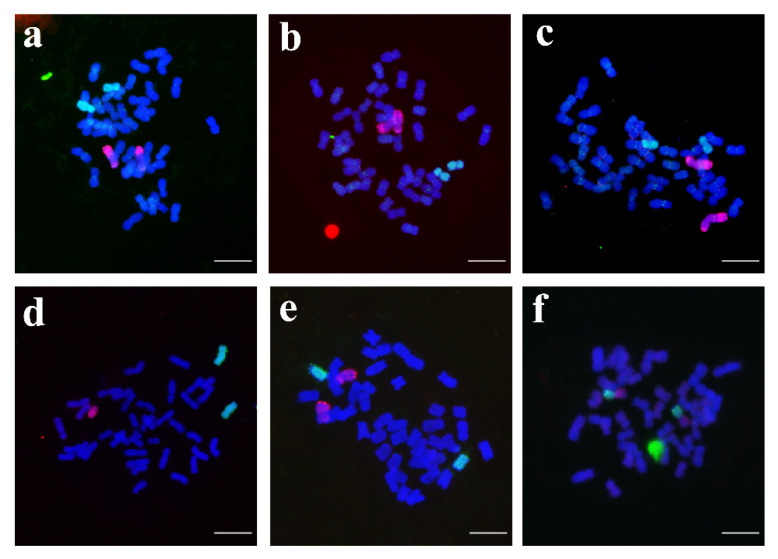
Multicolor GISH of six new breeding lines using *Th. elongatum* (4×) genomic DNA (green) and rye genomic DNA (red) as probes. Chromosomes were counterstained with DAPI (blue). (**a**) Line YZU006/1; (**b**) Line YZU006/5; (**c**) Line YZU035; (**d**) Line YZU047/1; (**e**) Line YZU047/2; (**f**) Line YZU048. Scale bar = 100 μm.

In this study, GISH was used for identification to determine the number of alien chromosomes and whether there was a translocation chromosome in the new breeding line. The results showed that the alien chromosome composition of the new breeding lines differed from that of YZU21, but all came from YZU21. Among them, four lines, YZU006/1, YZU006/5, YZU035, and YZU047/2, contained a pair of rye chromosomes and a pair of *Th. elongatum* chromosomes. Line YZU047/1 contains a pair of *Th. elongatum* chromosomes, and one rye chromosome. Line YZU48 contains a pair of translocated chromosomes. Cytological and PCR results showed that line YZU006/1 has 42 chromosomes, including 19 pairs of wheat chromosomes, 1 pair of 3E chromosomes, and 1 pair of 6R chromosomes (Figure 3a and Table 2); YZU006/5 has 44 chromosomes, including 20 pairs of wheat chromosomes, a pair of 2E chromosomes, and a pair of 6R chromosomes (Figure 3b and Table 2); YZU035 has 44 chromosomes, including 20 pairs of wheat chromosomes, a pair of 1E chromosomes, and a pair of 7R chromosomes (Figure 3c and Table 2); YZU047/1 has 42 chromosomes, including 39 wheat chromosomes, a pair of 5E chromosomes, and one 4R chromosome (Figure 3d and Table 2); YZU047/2 has 46 chromosomes, including 21 pairs of wheat chromosomes, a pair of 3E chromosomes, and a pair of 4R chromosomes (Figure 3e and Table 2); YZU048 has 42 chromosomes, including 20 pairs of wheat chromosomes and a pair of 3E/7R translocation chromosomes (Figure 3d and Table 2).

### 3.4. Evaluation of Disease Resistance

**Figure 4:**
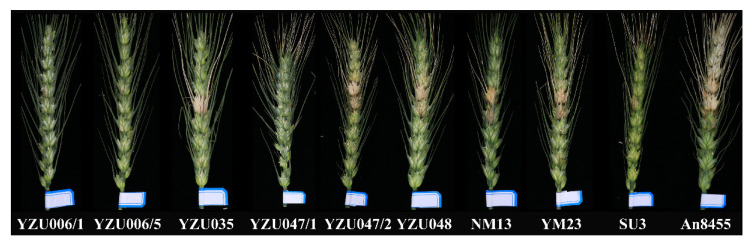
The phenotype of FHB resistance of six new breeding lines by single spikelet inoculation method at 21 after inoculation.

Six new lines were evaluated for FHB resistance and resistance to stripe rust and powdery mildew at the seedling stage. The results showed that all six lines were resistant to FHB, and the resistance level of YZU006/1, YZU006/5, YZU035, and YZU047/1 were similar to those of the resistant variety SU 3 (Figure 4 and Table 3). In addition, YZU006/5 was highly resistant to powdery mildew race E09 and moderately resistant to race A13 (Table 4). YZU006/1 and YZU006/5 showed high and moderate resistance, respectively, to race CYR32 of stripe rust (Table 4). Therefore, YZU006/5 is a new germplasm with resistance to FHB, powdery mildew, and stripe rust, and has potential application value in wheat disease resistance breeding.

## 4. Discussion

### 4.1. The Application of Related Species Is an Effective Approach to Wheat Genetic Improvement

Good varieties are not only an internal factor for obtaining high yield and quality of agricultural product, but also an important guarantee for world food security, seed industry security, and ecological security. In recent years, with the continuous improvement in technology, wheat breeding has made remarkable achievements in yield increase, stable yield, and high quality. However, the long-term hybridization and artificial selection of wheat varieties have led to the gradual narrowing of the scope of genetic variation in modern wheat varieties and the increasing simplification of many excellent resistance sources, such as stress resistance, insect resistance, and disease resistance, which has seriously restricted the further development of wheat breeding [26]. However, wheat relatives have retained many excellent traits that common wheat lacks in natural evolution, such as disease, insect, and drought resistance. Therefore, the transfer of excellent genes from wheat relatives to wheat through distant hybridization is an effective way to improve and broaden wheat genetic variations. For example, the 1RS chromosome of rye carries many genes related to high-yield, adaptability, and disease resistance, such as *Yr9*, *Sr31*, *Lr26*, *Pm8*, *Gb2,* and so on [27,28,29,30].

To date, many wheat varieties with high yields and good quality have been bred worldwide using wheat-rye 1BL/1RS translocation lines as parents. *Th. elongatum* also contained disease resistance genes, such as powdery mildew resistance gene *Pm51*, leaf rust resistance genes *Lr19*, *Lr2*, *Lr29*, stripe rust resistance gene *Yr69*, stem rust resistance genes *Sr24*, *Sr25*, *Sr26*, *Sr43,* and FHB resistance gene *Fhb7*. Some of these genes have been successfully transferred to common wheat and play important roles in the genetic improvement of wheat disease resistance [19,27,31,32]. For example, Wang et al. transferred chromosomal fragments carrying *Fhb7* to cultivated wheat varieties through distant hybridization combined with molecular marker-assisted selection. They revealed that *Fhb7* significantly improved wheat FHB resistance and had no negative impact on yield, indicating its potential application of *Fhb7* in wheat breeding for FHB resistance [19].

The excellent resistance to powdery mildew of *Haynaidia viiiosa* is due to the presence of many powdery mildew resistance genes in its genome, such as the *Pm67* gene on the 1VS chromosome [33], the *Pm62* gene on the 2VL chromosome [34], the 5VS chromosome carried *Pm55* gene [35], and the 6VS chromosome containing *Pm21* and *PmV* genes [36,37]. In addition to powdery mildew resistance genes, *Haynaldia villosa* genome also carries stripe rust, wheat yellow mosaic, leaf rust, stripe rust resistance genes, and other excellent genes that can improve wheat yield and quality [38,39]. Therefore, in the process of wheat breeding, scientists have created a series of special germplasms through distant hybridization, such as the translocation line T2VS·2DL that can increase spike length and grain number, the translocation line T6VS·6DL that can increase seed width and thousand kernel weight, and homozygous translocation lines T1AS·1AL-6VS and T4BS·4BL-6VS-4BL that carry the powdery mildew resistance gene *Pm21*, all of which have been used as intermediate germplasm in wheat breeding [40,41,42].

The P genome of *Agropyron cristatum* (AC) also carries excellent genes for disease resistance, stress resistance, and improving wheat yield. Therefore, breeders breed wheat AC 701 and AC 9946 with good spike characters, higher thousand kernel weight, and more grains per spike by using the wheat-*A. cristatum* 6P translocation line [43,44]. This showed that using distant crosses to transfer superior genes for disease resistance, stress tolerance, and yield increase from wheat relatives into wheat can enrich the genetic background of wheat and create new wheat germplasms with superior traits to meet the demand for superior wheat germplasm resources for modern breeding. In this study, the new breeding lines of *Triticum-Secale-Thinopyrum* trigeneric hybrids had desirable agronomic traits compared with the major wheat cultivars NM13 and YM23. For example, the spike length of YZU006/5, YZU047/1, YZU047/2, and YZU048 was significantly longer than NM13 and YM23; the thousand kernel weight of YZU035, YZU047/1, YZU047/2, and YZU048 was higher than that of NM13 and YM23. The grain length and width of YZU047/1 and YZU047/2 were significantly longer than those of NM13 and YM23. Therefore, these hybrid lines have higher yield potential for wheat breeding.

### 4.2. The Specific Molecular Marker and GISH Can Accurately Identify Trigeneric Hybrid

Accurate identification of alien chromosomes is the basis for enriching wheat germplasm resources using wheat-related species. However, it is difficult to identify alien chromosomes according to chromosome length, centromere position, arm ratio, or satellite characteristics. Therefore, developing more abundant chromosome identification techniques are highly significant for the efficient, rapid, and accurate identification of alien chromosomes and chromosome structure variation. However, in identifying alien chromosomes, there are some limitations to the single use of GISH, FISH, or molecular markers. For example, GISH can identify the alien chromosome or chromosome fragment on the wheat background but cannot distinguish the individual chromosome. Molecular markers can identify the individual chromosomes but cannot identify the number of chromosomes and the translocation of chromosomes. In the genome of higher plants, there are many repeat sequences in which tandem repeat sequences are mainly distributed in specific regions of chromosomes which are arranged in strings, and exist in clusters in the genome.

Therefore, developing tandem repeat sequences as probes for FISH analysis can help identify specific chromosomes according to the different positions of hybridization signals on chromosomes [45]. Recent studies have shown that when conducting FISH analysis, the signal produced by oligonucleotide probes was similar to that of traditional plasmid probes. Oligo-FISH has the advantages of simple operation steps, short time, and low cost of experimental design and use; thus, it has been widely used in identifying alien chromosomes in the context of wheat [46]. Generally, using oligo-FISH to identify alien chromosomes requires an accurate FISH karyotype; however, there are some differences in chromosome karyotypes between different wheat varieties, which affect the accuracy of alien chromosome identification. Therefore, combining different identification methods can improve the accuracy of alien chromosome identification.

For example, Dai et al. [20] identified the chromosome competition of *Triticum-Secale-Thinopyrum* trigeneric hybrids using molecular markers and GISH, and Ren et al. [47] identified the wheat-rye 6R, 6RS, and 6RL addition lines from hybrid progenies of wheat and rye using molecular markers and FISH. However, the composition of alien chromosomes in the previous generations after hybridization is typically unstable. Therefore, in this study, before the F_4_ and BC_1_F_2_ generations, only R and E genomic molecular markers were used to identify alien chromosomes, and combined with the agronomic characters, single plants with comprehensive shapes containing alien chromosomes were selected for harvest (Figure 1). From the F_5_ and BC_1_F_3_ generations, chromosome-specific molecular markers and genomic molecular markers were used together to identify alien chromosomes.

PCR results showed that YZU006/1 contained 3E and 6R chromosomes, YZU006/5 contained 2E and 6R chromosomes, YZU035 carried 1E and 7R chromosomes, YZU047/1 contained 4R and 5E chromosomes, YZU047/2 carried 3E and 4R chromosomes, YZU048 contained 3E and 7R chromosomes (Figure 2). However, chromosome-specific markers cannot identify the number of alien chromosomes and translocation chromosomes. Therefore, in this study, GISH was used to identify the alien chromosomes in addition to molecular marker identification. Combined with the PCR results, it was illustrated that two lines contained 44 chromosomes, including 20 pairs of wheat chromosomes, but the composition of alien chromosomes was different. Line YZU006/5 carried a pair of 2E and 6R chromosomes (Figure 3b and Table 2); line YZU035 contained a pair of 1E and 7R chromosomes (Figure 3c and Table 2). Although YZU006/1, YZU047/1, and YZU048 contained 42 chromosomes, the composition of these chromosomes was different. YZU006/1 carried 19 pairs of wheat chromosomes, a pair of 3E chromosomes, and a pair of 6R chromosomes (Figure 3a and Table 2), YZU047/1 only carried a pair of 5E chromosomes, as well as one 4R chromosome (Figure 3d and Table 2), whereas YZU048 carried 20 pairs of wheat chromosomes, and the translocation occurred between alien chromosomes, which was a pair of 3E/7R translocation chromosomes (Figure 3d and Table 2). In addition, YZU047/2 had 46 chromosomes, including 21 pairs of wheat chromosomes, a pair of 3E chromosomes, and a pair of 4R chromosomes (Figure 3e and Table 2). In summary, only molecular markers were used in the previous generations after hybridization in identifying alien chromosomes. When the agronomic characteristics of materials tend to be stable, identification by both molecular markers and GISH can reduce the workload of identification in the early stage and enable the accurate identification of the composition of alien chromosomes in the higher generation. If there is an accurate chromosome FISH karyotype map, the composition of alien and the composition of wheat chromosomes can be identified. Accurate identification of chromosome composition in these new breeding lines is beneficial for the better use of these germplasms in wheat breeding.

### 4.3. The novel Lines of Trigeneric Hybrid Provide New Parents in Wheat Breeding for Multiple Disease Resistance

To broaden the type of genetic variation in wheat and improve the genetic differences in wheat, scientists have gradually developed from using a single relative species and genus to comprehensively using multiple relative species and genera in the process of using wheat related species to improve wheat genetic variation, i.e., combining the genome or chromosome fragments of three or more genera to obtain hybrid germplasm with multiple and generic characteristics to meet the requirements of modern wheat breeding objectives. Moreover, it is possible to study the genetic relationship between different genomes in the same genetic background, which provides a basis for the evolutionary relationship between the genomes of *Triticeae* [48]. Trigeneric hybrid germplasms have been created, including wheat-rye-barley [49], wheat-rye-*Th. elongatum* [50], wheat-rye-*Leymus* [51], wheat-rye-*Agropyron* [52], and wheat-rye-*Aegilops* [53]; the new germplasm with high yield, disease, and pest resistance and strong stress resistance has been obtained on this basis.

For example, the trigeneric hybrid germplasm wheat-*Thinopyrum* intermedium-*Psathyrostachys huashanica* created by Kang et al. showed high resistance to *Pst* [54]. By crossing an octoploid wheat-*Th. intermedium* partial amphiploids with hexaploid triticale, Li et al. [55] produced new trigeneric hybrids with blue grains and resistance to wheat stripe rust. However, most of these new lines are resistant to only one disease. In a previous study, Dai et al. crossed T182 (AABBRR) with 8801 (AABBEE) to create trigeneric hybrid lines RE21 and RE62, which were both resistant to leaf rust and stem rust races Ug99; and RE21 also showed resistance to FHB [20]. However, RE21 still needs to be improved for some agronomic traits, such as plant height, thousand kernel weight and heading stage.

Therefore, in this study, to improve line RE21 and create new breeding lines with resistance to multiple wheat diseases under the background of common wheat, YZU21, a derivative of RE21, was used to cross with cultivated wheat NM13 and YM23, and six new breeding lines with stable agronomic characteristics were screened from the progenies of selfing or backcrossing. Among them, line YZU006/1 was resistant to FHB and stripe rust, and line YZU006/5 was resistant to FHB, powdery mildew, and stripe rust. Therefore, these two breeding lines have potential applications in wheat disease resistance breeding. Interestingly, we found that chromosome 6R was a common alien chromosome in these two lines.

Previous studies have found that the powdery mildew resistance gene *Pm20* is carried on the 6RL chromosome of Prolific rye [56], the *Pm56* gene was located on the 6RS chromosome of Qinling rye [6], and many rye varieties carrying powdery mildew resistance genes on 6RL chromosomes have been successfully used in wheat powdery mildew resistance breeding [30]. It has also been found that the 6R chromosome of rye also carries the stripe rust resistance gene [57]. For example, the stripe rust resistance gene *Yr83* is localized on chromosome 6RL of the triticale derivative T-701 [58], and a stripe rust resistance gene different from *Yr83* is found on chromosome 6RL of rye AR106BONE [59]. These findings suggest that both long and short arms of the 6R chromosome carry powdery mildew and stripe rust resistance genes [47]. In addition, the agronomic characteristics of these two lines were significantly improved compared with those of YZU21. For example, plant height was significantly reduced (Figure 1d), and the thousand-kernel weight of line YZU006/5 was significantly higher than that of YZU21 (Figure 1g). It follows that YZU006/1 and YZU006/5, created in this study, can be used as parents to improve wheat resistance to FHB, powdery mildew, and stripe rust. YUZ006/5 is more promising than YZU006/1 for wheat multiple disease resistance breeding applications.

## 5. Conclusions

In this study, YZU21 was crossed with cultivated wheat NM13 and YM23 to transfer resistance to multiple diseases in common wheat background. Specific molecular markers and GISH were used to identify hybrid progenies. Five addition or substitution lines and one translocation line of *Triticum-Secale-Thinopyrum* trigeneric hybrid were obtained. They showed desirable agronomic traits and improved resistance to FHB, powdery mildew, and stripe rust, which might be used as parents in wheat breeding for resistance to multiple diseases.

## Figures and Tables

**Table 1:** The hybrid combination and generation information of new lines.

Line	Hybrid Combination	Generation
YZU006/1	NM13/YZU21	F_6_
YZU006/5	NM13/YZU21	F_6_
YZU035	YM23//YM23/YZU21	BC_1_F_4_
YZU047/1	YM23//YM23/YZU21	BC_1_F_4_
YZU047/2	YM23//YM23/YZU21	BC_1_F_4_
YZU048	YM23//YM23/YZU21	BC_1_F_4_

**Table 2:** The alien chromosome and chromosome composition of new lines.

Line	Alien Chromosome	Chromosome Number	The Chromosome Composition
YZU006/1	3E and 6R	42	19″WW + 1″3E + 1″6R
YZU006/5	2E and 6R	44	20″WW + 1″2E + 1″6R
YZU035	1E and 7R	44	20″WW + 1″1E + 1″7R
YZU047/1	4R and 5E	42	19″WW +1′W + 1′4R + 1″5E
YZU047/2	3E and 4R	46	21″WW + 1″3E + 1″4R
YZU048	3E and 7R	42	20″WW + 1″3E/7R translocation

Note: ″ represents a pair of chromosomes and ′ represents a chromosome.

**Table 3:** Evaluation of resistance to FHB of new germplasm.

Line	Average Disease Spikelet Rate (%)
YZU006/1	7.7 ± 2.8 de
YZU006/5	9.9 ± 0.6 d
YZU035	7.9 ± 2.3 d
YZU047/1	9.6 ± 0.5 d
YZU047/2	16.1 ± 2.6 c
YZU048	13.1 ± 2.3 cd
NM 13	16.4 ± 2.4 c
YZU21	6.3 ± 2.4 de
YM 23	20.3 ± 3.0 b
SU 3	5.9 ± 2.5 e
An8455	87.2 ± 2.0 a

Note: The data were statistically analyzed via Kruskal-Wallis one-way ANOVA. Pairwise comparisons were completed using LSD. Different letters show significance at *p* < 0.05.

**Table 4:** Evaluation of resistance to powdery mildew and stripe rust of new lines.

Line	*Bgt*								*Pst*			
	E09 Race	ITs	E15 Race	ITs	A13 Race	ITs	A44 Race	ITs	CYR32 Race	ITs	CYR34 Race	ITs
YZU006/1	3	MS	4	HS	4	HS	3	MS	3	HR	7	MS
YZU006/5	0;	HR	4	HS	2	MR	4	HS	4	MR	7	MS
YZU035	4	HS	4	HS	4	HS	4	HS	7	MS	7	MS
YZU047/1	3	MS	4	HS	4	HS	4	HS	7	MS	7	MS
YZU047/2	4	HS	4	HS	4	HS	4	HS	7	MS	7	MS
YZU048	4	HS	3	MS	4	HS	4	HS	7	MS	7	MS
NM13	3	MS	4	HS	4	HS	3	MS	7	MS	7	MS
YZU21	0	I	0	I	4	HS	2	MR	7	MS	8	MS
YM23	4	HS	4	HS	4	HS	4	HS	2	HR	1	HR

Note: The infection type of powdery mildew: 0: Immunity (I), 0;: High resistance (HR), 1: High resistance (HR), 2: Moderate resistance (MR), 3: Moderate susceptible (MS), 4: Highly susceptible (HS). The infection type of stripe rust: 0–3: Resistance (R), 4–6: Moderate resistance (MR), 7: Moderate susceptible (MS), 8–9: Susceptible (S).

## Data Availability

Not applicable.

## Supplementary Materials

The following supporting information can be downloaded at: https://www.mdpi.com/article/10.3390/genes13122345/s1, Table S1: The sequences of the specific molecular markers for PCR used in this study.

## References

[1] Mayer K.F.X., Rogers J., Dolezel J., Pozniak C., Eversole K., Feuillet C., Gill B., Friebe B., Lukaszewski A.J., Sourdille P. (2014). A chromosome-based draft sequence of the hexaploid bread wheat (*Triticum aestivum*) genome. Science.

[2] Geiger H.H., Miedaner T., Carena M.J. (2009). Rye Breeding. Cereals.

[3] Heun M., Friebe B. (1990). Introgression of powdery mildew resistance from rye into wheat. Phytopathology.

[4] Hsam S.L.K., Zeller F.J. (1997). Evidence of allelism between genes *Pm8* and *Pm17* and chromosomal location of powdery mildew and leaf rust resistance genes in the common wheat cultivar ‘Amigo’. Plant Breed..

[5] Friebe B., Heun M., Tuleen N., Zeller F.J., Gill B.S. (1994). Cytogenetically monitored transfer of powdery mildew resistance from rye into wheat. Crop Sci..

[6] Hao M., Liu M., Luo J.T., Fan C.L., Yi Y.Y., Zhang L.Q., Yuan Z.W., Ning S.Z., Zheng Y.L., Liu D.C. (2018). Introgression of powdery mildew resistance gene *Pm56* on rye chromosome arm 6RS into wheat. Front. Plant Sci..

[7] Rao M.P., Ramanujam S. The transfer of alien genes for stem rust resistance to durum wheat. Proceedings of the International Wheat Genetics Symposium.

[8] Mago R., Miah H., Lawrence G.J., Wellings C.R., Spielmeyer W., Bariana H.S., McIntosh R.A., Pryor A.J., Ellis J.G. (2005). High-resolution mapping and mutation analysis separate the rust resistance genes *Sr31*, *Lr26* and *Yr9* on the short arm of rye chromosome 1. Theor. Appl. Genet..

[9] Mago R., Zhang P., Vautrin S., Šimková H., Bansal U., Luo M.C., Rouse M., Karaoglu H., Periyannan S., Kolmer J. (2015). The wheat *Sr50* gene reveals rich diversity at a cereal disease resistance locus. Nat. Plants.

[10] Rahmatov M., Rouse M.N., Nirmala J., Danilova T., Friebe B., Steffenson B.J., Johansson E. (2016). A new 2DS·2RL robertsonian translocation transfers stem rust resistance gene *Sr59* into wheat. Theor. Appl. Genet..

[11] Mcintosh R.A., Wellings C.R., Park R.F., Jeans K., Cloud-Guest A. (1995). The genes for resistance to stripe rust in wheat and triticale. Wheat Rusts: An Atlas of Resistance Genes.

[12] Ren T.H., Jiang Q., Sun Z.X., Zhao L.Q., Peng W.H., Ren Z.L., Tan F.Q., Luo P.G., Li Z. (2022). Development and molecular cytogenetic characterization of novel primary wheat-rye 1RS.1BL translocation lines from multiple rye sources with resistance to stripe rust. Plant Dis..

[13] Lafiandra D., Riccardi G., Shewry P.R. (2014). Improving cereal grain carbohydrates for diet and health. J. Cereal Sci..

[14] Góral T., Wiśniewska H., Ochodzki O., Walentyn-Góral D. (2016). Higher *Fusarium* toxin accumulation in grain of winter Triticale lines inoculated with *Fusarium culmorum* as compared with wheat. Toxins.

[15] Jauhar P.P., Peterson T.S., Xu S.S. (2009). Cytogenetic and molecular characterization of a durum alien disomic addition line with enhanced tolerance to Fusarium head blight. Genome.

[16] Shen X., Ohm H. (2006). Fusarium head blight resistance derived from *Lophopyrum elongatum* chromosome 7E and its augmentation with *Fhb1* in wheat. Plant Breed..

[17] Liu H.P., Dai Y., Chi D., Huang S., Li H., Duan Y.M., Cao W.G., Gao Y., Fedak G., Chen J.M. (2018). Production and molecular cytogenetic characterization of a durum Wheat-*Thinopyrum elongatum* 7E disomic addition line with resistance to Fusarium head blight. Cytogenet. Genome Res..

[18] Guo J., Zhang X.L., Hou Y.L., Cai J.J., Shen X.R., Zhou T.T., Xu H.H., Ohm H.W., Wang H.W., Li A.F. (2015). High-density mapping of the major FHB resistance gene *Fhb7* derived from *Thinopyrum ponticum* and its pyramiding with *Fhb1* by marker-assisted selection. Theor. Appl. Genet..

[19] Wang H.W., Sun S.L., Ge W.Y., Zhao L.F., Hou B.Q., Wang K., Lyu Z.F., Chen L.Y., Xu S.S., Guo J. (2020). Horizontal gene transfer of *Fhb7* from fungus underlies Fusarium head blight resistance in wheat. Science.

[20] Dai Y., Duan Y.M., Liu H.P., Chi D., Cao W.G., Xue A., Yong G., George F., Chen J.M. (2017). Molecular cytogenetic characterization of two *Triticum*-*Secale*-*Thinopyrum* trigeneric hybrids exhibiting superior resistance to Fusarium head blight, leaf rust, and stem rust race Ug99. Front. Plant Sci..

[21] Lei J., Zhou J.W., Sun H.J., Wan W.T., Xiao J., Yuan C.X., Karafiátová M., Doležel J., Wang H.Y., Wang X.E. (2020). Development of oligonucleotide probes for FISH karyotyping in *Haynaldia villosa*, a wild relative of common wheat. Crop J..

[22] Zhao R.H., Wang H.Y., Xiao J., Bie T.D., Cheng S.H., Jia Q., Yuan C.X., Zhang R.Q., Cao A.Z., Chen P.D. (2013). Induction of 4VS chromosome recombinants using the CS ph1b mutant and mapping of the wheat yellow mosaic virus resistance gene from *Haynaldia villosa*. Theor. Appl. Genet..

[23] Yang H., Zhong S.F., Chen C., Yang H., Chen W., Tan F.Q., Zhang M., Chen W.Q., Ren T.H., Li Z. (2021). Identification and cloning of a CC-NBS-NBS-LRR gene as a candidate of *Pm40* by integrated analysis of both the available transcriptional data and published linkage mapping. Int. J. Mol. Sci..

[24] Ren T.H., Sun Z.X., Ren Z.L., Tan F.Q., Luo P.G., Tang Z.X., Fu S.L., Li Z. (2020). Molecular and cytogenetic characterization of a Wheat-Rye 7BS.7RL translocation line with resistance to stripe rust, powdery mildew, and Fusarium head blight. Phytopathology.

[25] Xie C., Sun Q., Ni Z., Yang T., Nevo E., Fahima T. (2004). Identification of resistance gene analogue markers closely linked to wheat powdery mildew resistance gene *Pm31*. Plant Breed..

[26] Warburton M.L., Crossa J., Franco J., Kazi M., Trethowan R., Rajaram S., Pfeiffer W., Zhang P., Dreisigacker S., Ginkel M. (2006). Bringing wild relatives back into the family: Recovering genetic diversity in CIMMYT improved wheat germplasm. Euphytica.

[27] Luo P.G., Ren Z.L., Wu X.H., Zhang H.Y., Zhang H.Q., Feng J.A. (2006). Structural and biochemical mechanism responsible for the stay-green phenotype in common wheat. Chin. Sci. Bull..

[28] Qi W.L., Tang Y., Zhu W., Li D.Y., Diao C.D., Xu L.L., Zeng J., Wang Y., Fan X., Sha L.N. (2016). Molecular cytogenetic characterization of a new wheat-rye 1BL1RS translocation line expressing superior stripe rust resistance and enhanced grain yield. Planta.

[29] Li S.Q., Tang H.P., Zhang H., Mu Y., Lan X.J., Ma J. (2020). A 1BL/1RS translocation contributing to kernel length increase in three wheat recombinant inbred line populations. Czech J. Genet. Plant Breed..

[30] Spetsov P., Daskalova N. (2022). Resistance to pathogens in wheat-rye and triticale genetic stocks. J. Plant Pathol..

[31] Hou L.Y., Jia J.Q., Zhang X.J., Li X., Yang Z.J., Ma J., Guo H.J., Zhan H.X., Qiao L.Y., Chang Z.J. (2016). Molecular mapping of the stripe rust resistance gene *Yr69* on wheat chromosome 2AS. Plant Dis..

[32] Li H.J., Wang X.M. (2009). *Thinopyrum ponticum* and *Th. intermedium*: The promising source of resistance to fungal and viral diseases of wheat. J. Genet. Genom..

[33] Zhang R.Q., Xiong C.X., Mu H.Q., Yao R.N., Meng X.R., Kong L.R., Xing L.P., Wu J.Z., Feng Y.G., Cao A.Z. (2021). *Pm67*, a new powdery mildew resistance gene transferred from *Dasypyrum villosum* chromosome 1V to common wheat (*Triticum aestivum* L.). Crop J..

[34] Zhang R.Q., Fan Y.L., Kong L.N., Wang Z.J., Wu J.Z., Xing L.P., Cao A.Z., Feng Y.G. (2018). *Pm62*, an adult-plant powdery mildew resistance gene introgressed from Dasypyrum villosum chromosome arm 2VL into wheat. Theor. Appl. Genet..

[35] Zhang R.Q., Sun B.X., Chen J., Cao A.Z., Xing L.P., Feng Y.G., Lan C.X., Chen P.D. (2016). *Pm55*, a developmental-stage and tissue-specific powdery mildew resistance gene introgressed from *Dasypyrum villosum* into common wheat. Theor. Appl. Genet..

[36] Chen P.D., Qi L.L., Zhou B., Zhang S.Z., Liu D.J. (1995). Development and molecular cytogenetic analysis of wheat-*Haynaldia villosa* 6VS/6AL translocation lines specifying resistance to powdery mildew. Theor. Appl. Genet..

[37] Yildirim A., Jones S.S., Murray T.D., Line R.F. (2000). Evaluation of *Dasypyrum villosum* populations for resistance to cereal eyespot and stripe rust pathogens. Plant Dis..

[38] De Pace C., Snidaro D., Ciaffi M., Vittori D., Ciofo A., Cenci A., Tanzarella O.A., Qualset C.O., Mugnozza G.T.S. (2001). Introgression of *Dasypyrum villosum* chromatin into common wheat improves grain protein quality. Euphytica.

[39] Qi L., Cao M., Chen P., Li W., Liu D. (1996). Identification, mapping, and application of polymorphic DNA associated with resistance gene *Pm21* of wheat. Genome.

[40] Zhang R.Q., Hou F., Feng Y.G., Zhang W., Zhang M.Y., Chen P.D. (2015). Characterization of a *Triticum aestivum*-*Dasypyrum villosum* T2VS center dot 2DL translocation line expressing a longer spike and more kernels traits. Theor. Appl. Genet..

[41] Feng Z.Y., Song L., Song W.J., Qi Z.Q., Yuan J., Li R., Han H.M., Wang H.F., Chen Z.Y., Guo W.L. (2021). The decreased expression of *GW2* homologous genes contributed to the increased grain width and thousand-grain weight in wheat-*Dasypyrum villosum* 6VS center dot 6DL translocation lines. Theor. Appl. Genet..

[42] Chen P.D., You C.F., Hu Y., Chen S.W., Zhou B., Cao A.Z., Wang X.E. (2013). Radiation-induced translocations with reduced *Haynaldia villosa* chromatin at the *Pm21* locus for powdery mildew resistance in wheat. Mol. Breeding.

[43] Zhou S.H., Zhang J.P., Han H.M., Zhang J., Ma H.H., Zhang Z., Lu Y.Q., Liu W.H., Yang X.M., Li X.Q. (2019). Full-length transcriptome sequences of *Agropyron cristatum* facilitate the prediction of putative genes for thousand-grain weight in a wheat-*A. cristatum* translocation line. BMC Genom..

[44] Zhang J., Zhang J.P., Liu W.H., Wu X.Y., Yang X.M., Li X.Q., Lu Y.Q., Li L.H. (2016). An intercalary translocation from *Agropyron cristatum* 6P chromosome into common wheat confers enhanced kernel number per spike. Planta.

[45] Heslop-Harrison J.S., Olmo E., Redl C.A. (2000). RNA, genes, genomes and chromosomes: Repetitive DNA sequences in plants. Chromosomes Today.

[46] Du P., Zhuang L.F., Wang Y.Z., Yuan L., Wang Q., Wang D.R., Dawadondup, Tan L.J., Shen J., Xu H.B. (2017). Development of oligonucleotides and multiplex probes for quick and accurate identification of wheat and *Thinopyrum bessarabicum* chromosomes. Genome.

[47] Ren T.H., Sun Z.X., Hu Y.L., Ren Z.L., Tan F.Q., Luo P.G., Li Z. (2022). Molecular cytogenetic identification of new wheat-rye 6R, 6RS, and 6RL addition lines with resistance to stripe rust and powdery mildew. Front. Plant Sci..

[48] Zaharieva M., Monneveux P. (2006). Spontaneous hybridization between bread wheat (*Triticum aestivum* L.) and its wild relatives in Europe. Crop Sci..

[49] Fernández-Escobar J., Martin A. (1989). A self-fertile trigeneric hybrid in the Triticeae involving *Triticum*, *Hordeum*, and *Secale*. Euphytica.

[50] Yu C.J., Jia X., Hu S.Q., Zhuang J.J. (1994). Cytogenetics of the hybrids and their pollen plants of three genera *Triticum Secale* and *Thinopyrum*. Acta Genetica Sinica.

[51] Li X.F., Song Z.Q., Liu S.B., Gao J.R., Wang H.G. (2006). Cytogenetic study of a trigeneric (*Triticale* × *Trileymus*) hybrid. Euphytica.

[52] Gupta P.K., Fedak G. (2011). Intergeneric hybrids between × *Triticosecale* cv. *Welsh* (2n = 42) and three genotypes of *Agropyron intermedium* (2n = 42). Genome.

[53] Orellana J., Vazquez J.F., Carrillo J.M. (1989). Genome analysis in wheat-rye-*Aegilops caudata* trigeneric hybrids. Genome.

[54] Kang H.Y., Tang L., Li D.Y., Diao C.D., Zhu W., Tang Y., Wang Y., Fan X., Xu L.L., Zeng J. (2017). Cytogenetic study and stripe rust response of the derivatives from a wheat-*Thinopyrum intermedium*-*Psathyrostachys huashanica* trigeneric hybrid. Genome.

[55] Li J.B., Lang T., Li B., Yu Z.H., Wang H.J., Li G.R., Yang E.N., Yang Z.J. (2017). Introduction of *Thinopyrum intermedium* ssp *trichophorum* chromosomes to wheat by trigeneric hybridization involving *Triticum*, *Secale* and *Thinopyrum* genera. Planta.

[56] Friebe B., Jiang J., Raupp W.J., McIntosh R.A., Gill B.S. (1996). Characterization of wheat-alien translocations conferring resistance to diseases and pests: Current status. Euphytica.

[57] Johansson E., Henriksson T., Prieto-Linde M.L., Andersson S., Ashraf R., Rahmatov M. (2020). Diverse wheat-alien introgression lines as a basis for durable resistance and quality characteristics in bread wheat. Front. Plant Sci..

[58] Li J.B., Dundas L., Dong C.M., Li G.R., Trethowan R., Yang Z.J., Hoxha S., Zhang P. (2020). Identification and characterization of a new stripe rust resistance gene *Yr83* on rye chromosome 6R in wheat. Theor. Appl. Genet..

[59] Duan Y.L., Luo J., Yang Z.J., Li G.R., Tang Z.X., Fu S.L. (2022). The physical location of stripe rust resistance genes on chromosome 6 of rye (*Secale cereale* L.) AR106BONE. Front. Plant Sci..

