# Supplementary material for: Transfer of the Resistance to Multiple Diseases from a Triticum-Secale-Thinopyrum Trigeneric Hybrid to Ningmai 13 and Yangmai 23 Wheat Using Specific Molecular Markers and GISH
Source: Genes (Basel). 2022 Dec 12;13(12):2345. doi: 10.3390/genes13122345 (PMC9778474; doi:10.3390/genes13122345)
Supplement: Supplementary file 1 [file genes-13-02345-s001.zip › genes-2014315-supplementary.pdf]

Table S1: The sequences of the specific molecular markers for PCR used in this study.

Markers	Forward (5′-3′)	Reverse (5′–3′)	Chromosome
CSM1E-2	GCAAGCATAGTAAGGAGCAA	TAAGGTCGTCCCAACAGG	1E
CSM2E-14	CCAAACCCTAACAAATCG	CATGGCTAGTGTAGTTTATCTTC	2E
CSM3E-11	CTTTTTGGGATGCTTGACCG	CCTTACCTGCCTGAGTTTCC	3E
CMS4R-33	ACCACGAGGAGAAGTTTGCT	AGTAATCAATTGTTGGCCCTT	4R
CMS5E-6	AATTCCCACAAAGGTTCCAC	TATTGTAGGTCGCTCGCTTG	5E
CMS6R-17	GCAGGAGGGGATGTTATGG	TATGTTGCACCTGTATTCTCA	6R
CMS7R-30	GCTGCACTTGTCTTAATGGACG	GATTGGGTTTCCATGCCTCG	7R
CMS1R-26	GCATCCATGCACAAGGTAAAGT	TCTACCCCATTCTGTCACTCCA	1R-7R
SLAF2E-12	ATGGGGTTTATTCCGTTTGC	ATTGCTGGGTCATCTTGGG	1E-7E
